# Nutritional composition and staling properties of gluten‐free bread‐added fermented acorn flour

**DOI:** 10.1002/fsn3.3890

**Published:** 2023-12-26

**Authors:** Ayşe Levent, Kübra Aktaş

**Affiliations:** ^1^ Department of Gastronomy and Culinary Arts, School of Applied Sciences Karamanoğlu Mehmetbey University Karaman Turkey

**Keywords:** acorn, bread, chickpea yeast, fermentation, gluten‐free

## Abstract

The present study aimed to improve the nutritional quality of gluten‐free bread with the addition of acorn flour and to determine the characteristics of the final product. Formulations were adjusted with separately non‐fermented and two different fermented acorn flours at different levels (0, 15, 30, and 45%). The breads were assessed in terms of their chemical and physical properties, and their staling characterization was also estimated. Results showed that the fermentation of acorn flour before adding it to the formulation affected some chemical properties, and the addition of increasing amounts of acorn flour generally had a positive effect on the chemical composition. Furthermore, the highest protein, total phenolic content, Ca, K, Mg, Mn, and Fe values were obtained from breads, including fermented acorn flour with chickpea yeast (FAC). However, compared to non‐fermented acorn flour (AF), FAC and fermented acorn flour without chickpea yeast (FA) addition caused decreases in weight and volume of breads. Both crust L* and crumb L* values showed a significant reduction with increased acorn addition levels (from 71.88 and 77.22 to 42.26 and 41.15, respectively). The highest initial and final hardness values (*T*_*0*_ and *T*_*∞*_) were observed with FAC‐added samples, and Avrami exponent *n* was higher than 1 for only FAC‐added breads. Although fermented acorn addition had positive effects on the nutritional profile of breads, the sensory properties of the samples were negatively affected.

## Graphical abstract

Two different fermented acorn flours were incorporated into gluten‐free breads, and acorn flours generally improved the nutritional profile of bread samples. The use of chickpea yeast in the fermentation of acorn flour positively affected some chemical properties of breads, and lower weight and volume values were obtained with fermented acorn flours. Fermentation of acorn flours with chickpea yeast led to the lowest kinetic constant (k) as one of the staling properties.

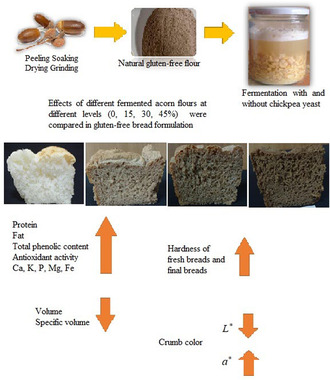

## 1 INTRODUCTION

Gluten is a protein that is available in cereal grains such as wheat, rye, and barley, and it generates a strong network for the desired structure in bakery products. Some people have a gluten intolerance called celiac disease (CD), and inflammation of the small intestine appears in this chronic disease. The patients lack the ability to absorb most of the nutrients that they consume. Although the prevalence of CD is reported as about 1% of the population, recent studies showed that along with undiagnosed “silent” or “latent” CD, this ratio increased. The best‐known treatment for this disorder is removing gluten from the diet throughout the patient's life (Diowksz & Sadowska, 2021; Krupa et al., 2010). In addition to celiac patients, the number of non‐celiac people adopting a gluten‐free diet for a healthier life has started to increase in recent years. Therefore, the market for gluten‐free products has expanded since gluten‐free products have been attracting considerable attention lately (da Costa Borges et al., 2021). However, the production of gluten‐free products still has both technological and nutritional disadvantages for food scientists and technologists. Especially gluten‐free bread has defects, including low volume, friable texture, poor flavor, and rapid firming, in addition to low protein, fiber, and mineral contents. Due to these problems, alternative flour sources are constantly being researched (Lancetti et al., 2022).

While many nutritional and structural improvement studies have been carried out even in breads using standard wheat flour (Atudorei and Codină, 2020; Coţovanu and Mironeasa, 2022; Dube et al., 2020), such studies have also become more important in gluten‐free breads. In gluten‐free bread formulations, rice flour is generally used due to its neutral flavor and pale color. Also, various alternative flours, including buckwheat, sorghum, quinoa, maize, teff, chia, amaranth, tigernut, chestnut, and legume flours, fruit and vegetable powders, and seafood powders, have been incorporated into the formulas. The combining of these flours and different starch sources, such as potato, corn, cassava, and tapioca has been examined in different studies. Besides these substitute flours and starches, functional additives, optimal processing methods, and some recent technologies, including transgenesis, enzymolysis, and fermentation, have been used to improve gluten‐free bread (Gao et al., 2018; Salehi, 2019; Sofi et al., 2023).

The acorn is a fruit of the oak tree, and acorn flour has been used in human nutrition for many years. Especially the Quercus (Q.) genus has been consumed by low‐income members of society as a dietary constituent up to the early twentieth century in Europe. It has been commonly used for bread‐making or as a coffee substitute. Because of its gluten‐free and nutritious composition, the utilization of acorn flour has recently been investigated in both gluten‐free and gluten‐containing formulas (Amina et al., 2018; Papoti et al., 2018; Sasani et al., 2023). Among the various species, the carbohydrate, fiber, protein, fat, and mineral values of acorn have been determined to be in the ranges of 41.52%–78.83%, 13.11%–51.76%, 2.08%–4.94%, 0.76%–3.08%, and 1.78%–3.21%, respectively (Skendi et al., 2022). Furthermore, acorn contains oleic (more than 50%), linoleic, and palmitic acids, besides containing phenolics and essential amino acids.

Shiri et al. (2021) used acorn flour and inulin with different fermentation types in rice‐based gluten‐free bread production, and they reported that gas restoration was obtained during the baking process with mixed fermentation based on sourdough with 10% long‐chain inulin and 30% acorn flour. On the other hand, a 50% addition of acorn to the formulation showed negative effects on the technological characteristics of bread samples. Beltrão Martins et al. (2020) incorporated acorn flour into gluten‐free bread formulation at 23% and 35% levels, and they revealed that acorn flour could be accepted as a good ingredient for bioactive compounds and antioxidants in gluten‐free bread, and the 23% incorporation level was approved in terms of sensory assessment. In another study, Beltrão Martins, Garzón, et al. (2022) reported that the combination of sourdough and acorn flour decreased the rate and extent of starch hydrolysis while increasing the mineral content, total phenolic compounds, and antioxidant activity.

Due to improver effects on bread quality and delaying effects on staling, sourdough utilization has been widely documented. Thanks to sourdough fermentation, bacteria produce organic acids, and as a result of this, acidification occurs and affects the dough rheology and bread flavor (Diowksz and Sadowska, 2021; Jagelaviciute and Cizeikiene, 2021). Sourdough fermentation can be obtained by spontaneous fermentation or accompanied by defined starter cultures and baker's yeast (Drakula et al., 2021). Besides, in some regions, such as the Mediterranean and Balkan countries, chickpea yeast has been used as a type of sourdough fermentation. The favorable changes regarding the sensorial, functional, nutritional, and textural quality of bakery products can be obtained with chickpea sourdough (Hendek Ertop & Coşkun, 2018). Șahin et al. (2018) added chickpea yeast to the gluten‐free bread formulations at 15, 30, and 45% (v/w) and achieved a loaf volume increment of about 3 and 12%, retarded staling, improved flavor, and increased nutritional quality by about 10%. Also, they inferred that the most suitable addition level was 30%.

This study aims to meet the nutritional deficit of gluten‐free bread thanks to acorn flour addition and to compare the different incorporation methods of acorn flour in gluten‐free bread making. Thus, acorn flour and fermented acorn flours with and without chickpea yeast were used at the different addition levels, and the enriched final products were evaluated for chemical characteristics and staling properties.

## 2 MATERIALS AND METHODS

### 2.1 Materials

The foodstuffs needed to prepare gluten‐free bread; rice flour, corn starch, bakers' yeast, salt, granulated sugar, baking powder, and sunflower oil were procured from local markets in Karaman, Turkey. Whole milk powder and guar gum were supplied from Enka Dairy Products Inc., Konya, Turkey, and Kimbiotek Chemical Substances Inc., Istanbul, Turkey, respectively. Chickpeas were purchased from a local legume retailer. Acorns (*Quercus ithaburensis*) were collected at the maturity stage from Konya, Turkey, and were soaked (1:50 g/mL) for debittering after their outer layers were taken away. Then, they were dried and turned into flour with a laboratory‐scale mill (Bosch, MKM600). The flour was stored in a refrigerator (4°C) until further use.

### 2.2 Fermentation of acorn flour

The modified method of Katina et al. (2007) was performed for the fermentation of acorn flour. Two different ways were pursued in the study. In the first fermentation treatment, 126 g of acorn flour, 5 g of bakers’ yeast, and 5 g of sugar were mixed with 201 g of water, and the mixture was fermented at 30°C for 20 h. In the second fermentation treatment, differently from the former, the chickpea yeast (as a filtrate obtained from the fermented mixture (at 40°C for 16 h) of coarsely grounded chickpea (100 g), salt (1 g), and water (350 mL) (Șahin et al., 2018)) were used instead of water.

### 2.3 Preparation of gluten‐free bread

The method of Yeşil and Levent (2022) was implemented for gluten‐free bread production. The recipe for the control gluten‐free bread contained 100 g gluten‐free flour (rice flour: corn starch, 50:50), 3 g bakers yeast, 1.5 g salt, 6 g sugar, 2 g baking powder, 5 g whole milk powder, 5 mL sunflower oil, 1 g guar gum, and 180 mL water. The ingredients were mixed in a mixer (Kitchen‐aid, Artisan Series, Greenville, OH, USA) at low speed for 10 min to obtain a uniform dough consistency. After that, the gluten‐free mix was placed in a metal pan and left to ferment. The fermentation was fulfilled in a chamber (Fimak FMD16, Konya, Turkey) for 30 min at 30°C with 85% relative humidity, and baking was carried out in an oven (Bosch HGD52D120T, Istanbul, Turkey) at 180°C for 25 min. Then bread samples were cooled for at least 1 h before experimental evaluation. For the production of all fortified samples, dry gluten‐free flour mix was replaced with acorn flour types based on dry weight at 0, 15, 30, and 45% levels. The bread experiments were performed for two independent variables: acorn flour type (powder, fermented with and without chickpea yeast) and acorn flour ratio (0, 15, 30, and 45%) by applying a (3 × 4) × 2 factorial design. The bread production was applied in duplicate, and mean values were displayed.

### 2.4 Chemical composition

The methods offered by the American Association of Cereal Chemists with method numbers 44‐19, 08‐01, 46‐12 and 30‐25 were used for the estimation of moisture, ash, crude protein, and crude fat contents, respectively (AACC, 1990).

The extraction process, total phenolic content (TPC), and antioxidant activity (AA) analyses were conducted according to a modified procedure by Wronkowska et al. (2010). The ground gluten‐free samples were prepared together with aqueous methanol (methanol/distilled water, 8:2, v/v) and shaken for 2 h at 30°C for extraction. The mixtures were centrifuged at 1600 *g* for 15 min, and the obtained fresh supernatants were used as extracts for the determination of both TPC and antioxidant capacity.

TPC was estimated with the Folin–Ciocalteu phenol reagent. The extract, diluted Folin–Ciocalteu reagent, sodium carbonate solution, and distilled water were mixed, and the mixtures were allowed to wait at room temperature in a dark place. The absorbance values of the samples were obtained with measurements at 765 nm using a spectrophotometer (Shimadzu UV1800, Japan). The values were given as mg of gallic acid equivalent (GAE) per 100 g dry‐weight basis.

On the other hand, the antioxidant activity of the extracts was measured against the 2,2 diphenyl‐1‐picrylhydrazyl (DPPH) radical. The extract, DPPH radical and solution, 80% methanol were mixed, and the mixtures were allowed to wait in the dark for 20 min. The blank consisted of a DPPH solution (250 μL) and aqueous methanol (2.1 mL). The data were collected by measurements at 517 nm using a spectrophotometer (Shimadzu UV1800, Japan). The inhibition was calculated as a percentage.

The mineral contents (Ca, K, P, Mg, Mn, and Fe) were detected with an inductively coupled plasma mass spectrometer (ICP–MS) (Agilent 7700 series; Agilent Technologies Tokyo, Japan). To perform the detection, dried samples were treated with sulfuric acid and kept for 24 h at room temperature. Hydrogen peroxide was used for discoloration, while sulfuric acid was added to the heated samples. The mineral values of samples were obtained from filtrates and displayed as mg/100 g.

### 2.5 Physical properties

The weight of gluten‐free bread was determined by the precision balance, and the seed displacement method was used for volume determination. The specific volume of bread was calculated by dividing the volume by the bread weight (Elgün et al., 2001).

The Hunter color values were obtained with a Minolta colorimeter (Konica CR 400, Osaka, Japan), where L* indicates whiteness (100)‐blackness (0), a* indicates red (+)‐green (−), and b* indicates yellow (+)–blue (−).

### 2.6 Staling characteristics

For hardness measuring of gluten‐free bread samples, a texture analyzer (TA‐XT2, Stable Microsystems, Surrey, UK) with a cylindrical probe of 25 mm in diameter and a 5 kg load cell was used. Measurements were conducted as the maximum force (g) with 25% compression height on bread slices with a width of 25 mm. The parameters, pre‐test speed, test speed, and post‐test speed, were 1.0 mm/s, 1.7 mm/s, and 5 mm/s, respectively.

Staling kinetics were determined by fitting hardness values obtained with along storage (0, 1, 2, and 3 days) according to Avrami equation (Equation 1), where *θ* is the fraction of recrystallization, *T*_*0*_, *T*_*∞*_, and *T*_*t*_ were hardness values at zero, infinite, and *t* times, respectively. k was the constant rate, and *n* was the Avrami exponent (Armero & Collar, 1998; Salinas & Puppo, 2018).

(1) $$\theta \;\left(t\right)=\left(T_{\infty }-T_{t}\right)/\left(T_{\infty }-T_{0}\right)=\exp\;\left(-k.t^{n}\right)$$

The crystallization half‐time (*t*_1/2_) is given as the time of half‐completed (50%) crystallization extent. It can be derived from the kinetic parameters k and *n*, according to Equation 2 (Salinas & Puppo, 2018).

(2) $$t_{1/2}={\left(\ln\;2/k\right)}^{1/n}$$

### 2.7 Sensory properties

A seven‐point hedonic scale (1 = dislike extremely; 4 = acceptable; 7 = like extremely) was applied in terms of color, pore structure, symmetry, taste, odor, and general acceptability for sensory analysis. The panel, consisting of 7 panelists from Karamanoğlu Mehmetbey University, carried out the analysis in an area far from the work area, unaffected by the preparation of samples. The gluten‐free breads produced in this study were sized evenly and presented to the panelists in a randomized order. In order not to mix the flavors, a glass of water was given to the panelists between the assessments of the samples.

### 2.8 Statistical analysis

Statistical analysis was conducted using the SPSS 20 statistical program (Version 22.0.;IBM Corp., Armonk, NY). The obtained data were subjected to analysis of variance, and Duncan's multiple range test was used to differentiate among the means. A significance level of 5% was adopted for all mean comparisons.

## 3 RESULTS AND DISCUSSION

### 3.1 Chemical properties of raw materials

The chemical composition of acorn flour, rice flour, and corn starch as basic ingredients of gluten‐free bread samples is compared in Table 1. Rice flour and corn starch had higher moisture contents than acorn flour. While the lowest ash (0.22 g/100 g) and protein (0.97 g/100 g) contents were obtained from corn starch, the highest ash (0.95 g/100 g) and protein (5.29 g/100 g) contents were obtained from acorn flour. However, acorn flour was found remarkable for its high fat, TPC, and AA. The results of these analyses were 9.82 g/100 g, 992.03 mg GAE/100 g, and 87.40%, respectively, for acorn flour. Polimac and Koceva Komlenić (2016) presented the moisture, ash, fat, and protein contents of native and thermally treated acorn flour with the following results: 9.45–3.51, 1.86–2.06, 4.44–4.67, and 6.35–6.61 g/100 g, respectively. Beltrão Martins, Garzón, et al. (2022) reported that moisture, protein, fat, and ash contents of acorn flour were 9.8, 4.8, 9.8, and 1.6 g/100 g, respectively. Another study indicated that the acorn flour had the highest fat content (11.39%) compared with other common flours (e.g., rice, maize, sorghum, and buckwheat) and 16.79 mg GAE/g total phenols (Beltrão Martins, Gouvinhas, et al., 2022). Also, Vinha et al. (2016) investigated various organs and tissues of four different *Quercus* species. They obtained values for the total phenolic compounds and DPPH radical scavenging activity as 18–32 mg GAE/g and 42%–74%, respectively. It was stated in the literature that the major phenolic compounds of acorns are gallic acid derivatives (such as methyl gallate, galloyl derivatives, galloyl methyl gallate, and tetragalloyl‐hexoside) together with tannins, and these phenolic compounds are known to be important in protecting health. Also, it was noted that acorns and acorn oil can be quantitatively compared to prominent phenolic sources in the diet, such as extra virgin olive oil (Szabłowska & Tańska, 2021).

**TABLE 1:** Chemical composition of raw materials.

	Acorn flour	Rice flour	Corn starch
Moisture (g/100 g)	5.20 ± 0.08^b^	9.24 ± 0.04^a^	9.36 ± 0.01^a^
Ash (g/100 g)	0.95 ± 0.01^a^	0.73 ± 0.03^b^	0.22 ± 0.01^c^
Protein (g/100 g)	5.29 ± 0.10^a^	3.82 ± 0.04^b^	0.97 ± 0.08^c^
Fat (g/100 g)	9.82 ± 0.05^a^	0.93 ± 0.13^b^	0.55 ± 0.06^c^
TPC (mg GAE/100 g)	992.03 ± 6.19^a^	17.11 ± 0.32^b^	9.22 ± 0.47^b^
AA (Inhibition %)	87.40 ± 1.15^a^	7.75 ± 0.07^b^	2.12 ± 0.81^c^
Ca (mg/100 g)	48.01 ± 0.34^a^	15.99 ± 0.16^c^	18.46 ± 0.12^b^
K (mg/100 g)	505.80 ± 3.45^a^	132.09 ± 0.98^b^	6.36 ± 0.24^c^
P (mg/100 g)	309.60 ± 3.00^a^	239.87 ± 2.58^b^	171.54 ± 0.47^c^
Mg (mg/100 g)	30.07 ± 0.16^a^	28.72 ± 0.22^b^	6.29 ± 0.01^c^
Mn (mg/100 g)	0.11 ± 0.00^b^	0.70 ± 0.01^a^	0.07 ± 0.01^c^
Fe (mg/100 g)	0.76 ± 0.03^a^	0.38 ± 0.03^b^	0.12 ± 0.01^c^

*Note*: Results are based on dry matter. The values followed by different lower case letter within a raw are significantly different (*p* ≤ .05).

Abbreviations: AA, Antioxidant activity; TPC, Total phenolic content.

The mineral contents (Ca, K, P, Mg, Mn, and Fe) of raw materials are shown in Table 1. All of the mineral contents except Mn of acorn flour were higher among others. Especially K content was quite higher than the others. Ca, K, P, Mg, Mn, and Fe contents were found to be 48.01, 505.8, 309.6, 30.07, 0.11, and 0.76 mg/100 g, respectively. Beltrão Martins, Gouvinhas, et al. (2022) obtained a similar result, and they found that K was the most abundant mineral in acorn flour, with 697.10 mg/100 g. Also, they noted down other minerals as Ca 51.66, P 81.56, Mg 65.82, Mn 7.78, and Fe 0.81 mg/100 g. Rakić et al. (2006) reported the values as follows: Ca 0.10%, K 0.83%, P 0.10%, Mg 0.04%, Mn 3 mg/kg, and Fe 41 mg/kg. It is possible to understand that the differences in the chemical composition of acorn flour can be due to different *Quercus* spices. It is known that, along with higher total phenolic content and antioxidant activity, products including acorn flour have a higher mineral content. Considering mineral deficiencies in diets, acorn usage may promote the bioavailability of minerals (Szabłowska & Tańska, 2021). Thus, acorn flour has recently been more scrutinized because of its good nutritional profile among other ingredients used in gluten‐free bakery product formulations.

### 3.2 Chemical properties of gluten‐free bread samples

The chemical properties of gluten‐free bread samples are given in Table 2. The moisture contents of fermented acorn flours added to bread were higher than those of non‐fermented acorn flour (AF) added samples, and the ash content of bread changed between 2.73 and 2.99 g/100 g. However, there was no statistically significant (*p* < .05) difference. Concerning addition levels, the control sample revealed lower moisture and ash contents (61.83 and 2.44 g/100 g, respectively) than others, and these values were 63.51 and 3.19 g/100 g, respectively, at 45% addition level. On the other hand, using FAC increased the protein, fat content, and TPC of bread. In order of protein, fat, and TPC analysis, AF, FA, and FAC‐added samples had 4.87–5.13–5.35 g/100 g, 3.38–4.01–4.16 g/100 g, and 1.18–1.32–1.51 mgGAE/g values, respectively. It was considered that the proteins could cross from chickpea to chickpea yeast, and this could be reflected in the bread composition. Korus et al. (2015) applied partial replacement in a gluten‐free formulation by debiting acorn flour. It was reported that 1.22, 2.07 g/100 g, and 1.88, 2.80 g/100 g values were observed with control and 40% FA‐added bread for protein and fat contents, respectively. The highest AA value was obtained with FA‐added samples (77.67%). As the substitution levels increased, the results showed that protein, fat contents, TPC, and AA values increased regularly, and remarkable changes occurred in TPC and AA values (from 0.15 mgGAE/g and 35.82% to 2.90 mgGAE/g and 96.61%, respectively). Especially, the antioxidant activity‐increasing effect of acorn flour was also observed in other studies (Parsaei et al., 2018). On the other hand, incorporating sourdough into gluten‐free formulations improves the nutritional profile. Also, the bioavailability and concentration of bioactive compounds come from the used ingredients, and LAB metabolism increases during sourdough fermentation. It was explained in the literature that, AF could be a substrate for LAB, and this might result in more metabolite production and the revealing of more phenolic compounds (Beltrão Martins, Garzón, et al., 2022). Also, it could be commented that acorn tannins transformation to phenols along fermentation increased phenol content (Nkhata et al., 2018). In this study, especially the fermentation process with chickpea yeast had an important effect on TPC. This situation can be explained by the diversity of LAB strains between sourdoughs and their different metabolisms (Beltrão Martins, Garzón, et al., 2022). Also, it is thought that during the preparation of chickpea yeast, the phenolic substances in chickpea may pass into the chickpea yeast liquid and cause an increase in the total phenolic content of the breads added.

**TABLE 2:** Chemical properties of gluten‐free samples supplemented with acorn flour.

	Moisture (g/100 g)	Ash (g/100 g)	Protein (g/100 g)	Fat (g/100 g)	TPC (mg GAE/g)	AA (inhibition %)
*Acorn flour addition types*						
AF	61.07 ± 0.39^b^	2.73 ± 0.29^a^	4.87 ± 0.76^c^	3.38 ± 0.99^b^	1.18 ± 1.02^c^	75.38 ± 24.06^b^
FA	64.15 ± 1.31^a^	2.75 ± 0.25^a^	5.13 ± 0.67^b^	4.01 ± 1.47^a^	1.32 ± 1.08^b^	77.67 ± 26.48^a^
FAC	63.47 ± 1.08^a^	2.99 ± 0.48^a^	5.35 ± 0.91^a^	4.16 ± 1.32^a^	1.51 ± 1.29^a^	76.98 ± 26.40^ab^
*Addition levels*						
0%	61.83 ± 1.04^b^	2.44 ± 0.06^c^	3.98 ± 0.21^d^	2.28 ± 0.20^d^	0.15 ± 0.01^d^	35.82 ± 1.87^d^
15%	62.87 ± 1.31^a^	2.69 ± 0.27^bc^	5.06 ± 0.28^c^	3.37 ± 0.38^c^	0.62 ± 0.13^c^	84.64 ± 1.63^c^
30%	63.39 ± 1.75^a^	2.97 ± 0.25^ab^	5.51 ± 0.31^b^	4.33 ± 0.59^b^	1.68 ± 0.14^b^	89.63 ± 1.23^b^
45%	63.51 ± 2.15^a^	3.19 ± 0.26^a^	5.92 ± 0.24^a^	5.41 ± 0.71^a^	2.90 ± 0.35^a^	96.61 ± 2.65^a^

*Note*: The values followed by different lower case letters within a column are significantly different (*p* ≤ 0.05).

Abbreviations: AA, Antioxidant activity; AF, Acorn flour; FA, Fermented acorn flour without chickpea yeast; FAC, Fermented acorn flour with chickpea yeast; TPC, Total phenolic content.

### 3.3 Mineral composition of gluten‐free bread samples

The mineral values of gluten‐free bread samples are presented in Table 3. As it can be seen from the table, the fermentation process affected the amount of minerals, and while all of the mineral contents except P were higher in the bread‐added FAC, only the P content was richer than others in FA‐containing bread. However, there was not a significant difference between the K and Mg results of AF and FA‐added bread. This mineral increment with FAC, different from other acorn addition types, may arise from the rich mineral content of chickpea yeast. When the addition ratios of different acorn flour types were examined, all of the minerals except Mn increased as the acorn ratio increased. Compared with the 0% substitution level, 1.21, 2.21, 1.26, 1.35, and 5.72 fold increases at the 45% substitution level were observed for Ca, K, P, Mg, and Fe contents, respectively. The most abundant minerals in bread were P and K, and their amounts ranged from 375.94 and 202.35 mg/100 g to 475.93 and 448.90 mg/100 g, respectively. In this case, it can be thought that acorn flour is a good mineral source compared to other raw materials, which affects the mineral content of the breads (Table 1). Furthermore, it was mentioned that fermentation increased some mineral contents, such as Mg, Fe, Ca, and Zn, in some fermented foods, and this was associated with the degradation of oxalates and phytates that attach to minerals (Nkhata et al., 2018). Beltrão Martins et al. (2020) produced gluten‐free bread with 35% AF replacement, and they displayed that usage of acorn flour contributed to an increase in K, Ca, and Mn contents.

**TABLE 3:** Mineral values of gluten‐free samples supplemented with acorn flour (mg/100 g).

	Ca	K	P	Mg	Mn	Fe
*Acorn flour addition types*						
AF	71.12 ± 4.42^c^	301.38 ± 79.68^b^	396.97 ± 20.15^c^	22.86 ± 2.02^b^	0.20 ± 0.03^c^	0.32 ± 0.21^b^
FA	74.27 ± 6.05^b^	301.08 ± 80.48^b^	452.15 ± 60.02^a^	23.24 ± 1.80^b^	0.21 ± 0.03^b^	0.29 ± 0.17^c^
FAC	75.03 ± 6.57^a^	376.81 ± 137.30^a^	429.98 ± 42.65^b^	26.93 ± 5.21^a^	0.29 ± 0.03^a^	0.42 ± 0.24^a^
*Addition levels*						
0%	65.93 ± 0.30^d^	202.35 ± 1.76^d^	375.94 ± 4.32^d^	20.74 ± 0.39^d^	0.26 ± 0.01^a^	0.11 ± 0.01^d^
15%	71.78 ± 2.73^c^	282.51 ± 27.99^c^	406.47 ± 13.47^c^	22.98 ± 1.34^c^	0.23 ± 0.03^b^	0.26 ± 0.09^c^
30%	76.29 ± 1.80^b^	371.92 ± 52.52^b^	447.12 ± 36.80^b^	25.53 ± 2.50^b^	0.23 ± 0.05^b^	0.39 ± 0.05^b^
45%	79.89 ± 3.39^a^	448.90 ± 74.99^a^	475.93 ± 45.62^a^	28.12 ± 4.42^a^	0.22 ± 0.08^b^	0.63 ± 0.10^a^

*Note*: The values followed by different lower case letters within a column are significantly different (*p* ≤ .05).

Abbreviations: AF, Acorn flour; FA, Fermented acorn flour without chickpea yeast; FAC, Fermented acorn flour with chickpea yeast.

### 3.4 Physical properties of gluten‐free bread samples

Weight, volume, and specific volume results belonging to gluten‐free bread are shown in Table 4. Usage of both FA and FAC adversely affected the results, especially FAC usage, which caused a notable reduction. Weight and volume values of AF, FA, and FAC‐added samples were recorded as 124.38, 122.50, 116.31 g, and 262.37, 238.37, and 199.38 mL, respectively. Over‐fermentation of breads added FAC by different bacteria, which may be available in chickpea yeast as different from other samples, can cause fermentation losses. Therefore, higher weight and volume losses in FAC‐added samples may be observed. Besides this, a significant decrease in bread volume was obtained by increasing enrichment ratios, and consequently, specific volume results decreased and were acquired between 1.61 and 2.30 mL/g. Korus et al. (2015) used AF in gluten‐free bread at 20, 40, and 60% levels, and they observed that the highest weight (202.7 g), lowest volume (407.0 cm^3^) and specific volume (2.01 cm^3^/g) were obtained with 60% AF‐added samples. The lower volume results were associated with increased water binding and/or changes in density, which resulted in obstructed proofing. We thought that the different densities of chickpea yeast might also contribute to the results obtained in this study. Consistent results were found in the study of Park et al. (2017); lower volume and specific volume were observed at the 25% AF addition level, but there was not a significant difference in weight values.

**TABLE 4:** Physical properties of gluten‐free bread samples supplemented with acorn flour.

	Weight (g)	Volume (mL)	Specific volume (mL/g)
*Acorn flour addition types*			
AF	124.38 ± 1.75^a^	262.37 ± 24.20^a^	2.10 ± 0.21^a^
FA	122.50 ± 1.67^b^	238.37 ± 34.02^b^	1.94 ± 0.29^b^
FAC	116.31 ± 3.52^c^	199.38 ± 50.11^c^	1.70 ± 0.38^c^
*Addition levels*			
0%	122.10 ± 1.06^a^	281.66 ± 1.21^a^	2.30 ± 0.01^a^
15%	119.42 ± 5.07^b^	235.00 ± 48.64^b^	1.94 ± 0.33^b^
30%	120.14 ± 5.28^b^	218.33 ± 39.54^c^	1.80 ± 0.26^c^
45%	122.60 ± 4.40^a^	198.50 ± 27.02^d^	1.61 ± 0.17^d^

*Note*: The values followed by different lower case letters within a column are significantly different (*p* ≤ .05).

Abbreviations: AF, Acorn flour; FA, Fermented acorn flour without chickpea yeast; FAC, Fermented acorn flour with chickpea yeast.

Using fermented or non‐fermented AF commonly influences the color properties of bread. As expected, the enriched bread had a darker color (Table 5). When the crust color characteristics were evaluated, the lowest L* (49.75) and highest a* (5.68) values were obtained with FAC‐added samples. When the crumb color characteristics were evaluated, the lowest L* (51.07) and highest a* (5.77) and b* (20.75) values were obtained with AF‐added samples. Sayaslan and Şahin (2018) used chickpea yeast in whole‐grain wheat flour bread production, and they reported that although fermented‐chickpea liquor addition did not have any significant effect on crumb color, it significantly reduced crust lightness (L*) and yellowness (b*). This case was explained by the effects of promoted caramelization and Maillard reactions with chickpea yeast, which contains sugar and free amino acids. When the addition levels were compared for both crust and crumb color, L* values tended to decrease, and a* values tended to increase. Similar trends were observed with lower levels (0, 0.5, 1, 3, 5%) in acorn‐enriched biscuits for L*and a* values (Joo et al., 2013). Also, there was not a significant difference among the b* values of acorn‐added bread.

**TABLE 5:** Color properties of gluten‐free samples supplemented with acorn flour.

	Crust color			Crumb color		
	L*	a*	b*	L*	a*	b*
*Acorn flour addition types*						
AF	57.11 ± 7.09^a^	4.33 ± 2.71^b^	21.36 ± 2.84^a^	51.07 ± 15.77^b^	5.77 ± 4.85^a^	20.75 ± 7.26^a^
FA	55.62 ± 13.47^a^	4.41 ± 3.28^b^	19.45 ± 2.91^a^	53.04 ± 17.00^a^	4.94 ± 4.33^b^	19.65 ± 6.36^b^
FAC	49.75 ± 15.14^b^	5.68 ± 4.07^a^	21.97 ± 5.09^a^	54.29 ± 13.28^a^	5.15 ± 4.29^b^	18.84 ± 6.33^c^
*Addition levels*						
0%	71.88 ± 3.76^a^	0.01 ± 0.56^c^	18.97 ± 5.77^a^	77.22 ± 3.07^a^	−1.82 ± 0.12^d^	9.01 ± 0.41^b^
15%	53.78 ± 3.36^b^	4.94 ± 1.47^b^	21.50 ± 4.45^a^	48.03 ± 1.47^b^	6.44 ± 0.55^c^	23.13 ± 1.49^a^
30%	48.72 ± 5.39^c^	5.74 ± 0.69^b^	21.39 ± 1.17^a^	44.79 ± 2.63^c^	7.77 ± 0.61^b^	23.77 ± 0.93^a^
45%	42.26 ± 7.52^d^	8.53 ± 1.48^a^	21.85 ± 2.07^a^	41.15 ± 2.61^d^	8.77 ± 0.65^a^	23.07 ± 1.17^a^

*Note*: The values followed by different lower case letters within a column are significantly different (*p* ≤ .05).

Abbreviations: AF, Acorn flour; FA, Fermented acorn flour without chickpea yeast; FAC, Fermented acorn flour with chickpea yeast.

### 3.5 Staling properties of gluten‐free bread samples

The internal structure of bread is crucial for consumer acclaim, which becomes even more prominent when the structural difficulties of gluten‐free products are considered, and besides this, the textural changes may be followed for ascertaining the commercial useful life of the product (Arp et al., 2020). The hardness values of bread at different days were determined, and crumb staling properties are shown in Table 6. The initial and final hardness values of samples changed between 499.65–852.85 g and 1219.74–1697.15 g, respectively, for different acorn addition methods. The highest *T*_*0*_ and *T*_*∞*_ values were found for FAC‐added bread. The lowest bread volumes were obtained with the same bread (Table 4) and Yeşil and Levent (2022) also pointed out this relationship. Moreover, both hardness values increased with increased addition levels of acorns. As mentioned before, increased water binding and/or changes in density may cause this situation with the increase in acorn addition in *T*_*0*_ and *T*_*∞*_ values. Although there are many parameters that affect staling, the Avrami model is commonly used to find out staling kinetics based on starch retrogradation (Armero & Collar, 1998; Salinas & Puppo, 2018). It was reported in the literature that an exponent value of *n* = 1 represents a rod‐like growth from instantaneous nucleil *n* = 2 represents a rod‐like growth from sporadic nuclei, and *n* = 3 represents a crystal growth in the form of discs from sporadic nuclei (Mclver et al., 1968; Salinas & Puppo, 2018). The obtained n values in this study were lower than 1 (*n* < 1) for AF and FA‐added bread and higher than 1 (*n* > 1) for FAC‐added bread. Șahin et al. (2018) used chickpea yeast in gluten‐free breads and they reported that due to the fact that rice starch is highly prone to retrogradation, the staling‐retarding effect of chickpea yeast may have been limited. The exponent value seemed only higher than 1 for the 15% acorn addition level. The kinetic constant (k) values changed between 0.42 (for FAC‐added samples) – 0.84 (for FA‐added samples), and the highest k values were observed for control bread (0% addition level). As the AF rate increased in the formulation, k values showed an increase (0.59). Rosell and Santos (2010) reported that bread‐added pectin gave the lowest constant k. However, resistant starch and fiber blend showed the highest values of k and it was reported that dietary fibers from different origins mostly changes the qualitative and quantitative thermal profile on starch gelatinisation and amylopectin retrogradation and kinetics during storage in different extend depend on the fiber source. Moreover, FA‐added samples had the lowest *t*_1/2_ value. The various addition rates of acorn flour presented *t*_1/2_ values between 1.24 and 1.28, which were higher than the control value (0.91).

**TABLE 6:** Staling kinetic parameters of gluten‐free bread.

	*T*_*0*_	*T*_*∞*_	*n*	k	*t*_1/2_	*r* ^2^
*Acorn flour addition types*						
AF	580.58	1432.45	0.92	0.60	1.18	0.98
FA	499.65	1219.74	0.73	0.84	0.77	0.93
FAC	852.85	1697.15	1.07	0.42	1.61	0.99
*Addition levels*						
0%	363.82	1040.39	0.72	0.74	0.91	0.96
15%	455.63	1172.98	1.18	0.52	1.27	0.99
30%	655.69	1569.42	0.97	0.55	1.28	0.99
45%	1102.32	2016.33	0.73	0.59	1.24	0.98

*Note*: *T*_*0*_: hardness of fresh bread, *T*_*∞*_: final bread hardness, *n*: Avrami exponent, k: constant rate, *t*_1/2_: crystallization half‐time.

Abbreviations: AF, Acorn flour; FA, Fermented acorn flour without chickpea yeast; FAC, Fermented acorn flour with chickpea yeast.

### 3.6 Sensory properties of gluten‐free bread samples

**FIGURE 1:**
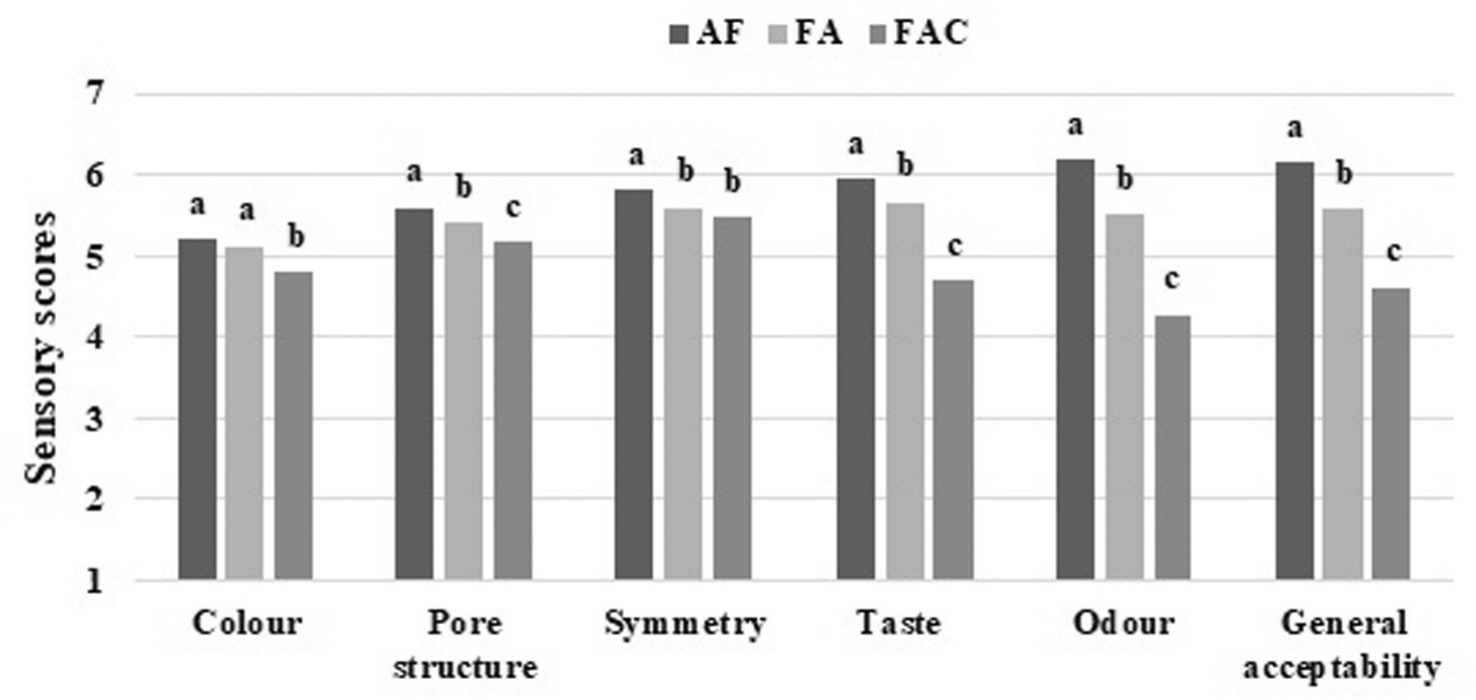
Sensory scores of gluten‐free bread samples. The values followed by different lower case letters are significantly different (*p* ≤ .05). AF, Acorn flour; FA, Fermented acorn flour without chickpea yeast; FAC, Fermented acorn flour with chickpea yeast.

The sensory evaluation of gluten‐free breads is displayed in Figure 1. The scores of samples for color, pore structure, symmetry, taste, odor, and general acceptability changed in the ranges of 4.80–5.20, 5.18–5.60, 5.48–5.82, 4.71–5.95, 4.28–6.20, and 4.62–6.15, respectively. Therefore, it can be said that the scores were above 4 (its meaning was acceptable), but the sensory properties of the gluten‐free breads have been adversely affected by the fermentation of acorn flour. The FAC‐added samples got generally lower scores than the others for all criteria except symmetry, and the most preferred samples were the ones with AF commonly. Besides this, when the results were examined in terms of addition levels in all of the criteria, the scores decreased steadily as the level increased from 0% to 45% (color, pore structure, symmetry, taste, odor, and general acceptability scores varied from 6.98, 6.36, 6.50, 6.73, 6.97, 6.95, to 3.28, 4.48, 4.41, 4.21, 4.15, 4.10, respectively; data not shown). Shiri et al. (2021) used acorn flour (10, 30, 50%) and inulin with different fermentation types in rice‐based gluten‐free bread production, and they expressed that improvement was observed in flavor and color perception at the 30% acorn flour replacement ratio, but its higher inclusion level adversely changed the color, flavor, texture, and consequently the overall acceptability. Saad et al. (2015) used 1.5, 3.0, and 4.5% chickpea steep liquor in leavened bread production, and they pointed out a decrease in the acceptability of the chickpea steep liquor‐fermented breads compared with the control. They stated that this was due to the fact that chickpea yeast resembled egg yolk, which could affect taste and color.

## 4 CONCLUSION

In this study, fermented and non‐fermented acorn flours were used in gluten‐free bread production separately. The results showed that the fermentation of acorn led to the development of nutritional content. The moisture, protein, fat, TPC, Ca, P, and Mn contents of fermented acorn‐added bread were higher compared with non‐fermented acorn flour‐added bread. Furthermore, the highest protein and fat content, TPC and AA (5.92%, 5.41%, 2.90 mg GAE/g, and 96.61%, respectively), were observed with a 45% substitution level. When the physical properties of breads were evaluated, the fermentation of acorn negatively affected the physical properties of breads. Especially using of chickpea yeast fermentation led to decrement in weight, volume, and specific volume measurements. Also, for both crust and crumb color, decreased L* and increased a* values were obtained depending on the acorn flour concentration. Besides these, the higher initial and final hardness values were measured in samples with FAC, and the staling kinetic parameters were determined using the Avrami model. For FAC‐used bread, the exponent n indicated a value which was higher than 1, and the lowest kinetic constant (k) was determined. On the other hand, the crystallization half‐time (*t*_1/2_) value of 0% added acorn flour was found to be lower than the others. According to sensory evaluation results, although the addition of fermented acorn flours contributed to the nutritional profile of gluten‐free breads, it was observed that these bread samples commonly negatively affected sensory. As a result, further studies can be conducted with FAC ratios not exceeding 30% and in the presence of some strains that promote fermentation under more controlled conditions.

## AUTHOR CONTRIBUTIONS

**Ayşe Levent:** Data curation (lead); formal analysis (lead); writing – original draft (equal). **Kübra Aktaş:** Data curation (supporting); formal analysis (supporting); project administration (lead); supervision (lead); writing – original draft (equal); writing – review and editing (lead).

## FUNDING INFORMATION

This article is taken from Ayşe LEVENT's master thesis supported by The Scientific Research Projects Coordination Unit of Karamanoğlu Mehmetbey University with the grant number 05‐YL‐21.

## CONFLICT OF INTEREST STATEMENT

The authors declare no conflict of interest.

## ETHICS STATEMENT

In the sensory evaluation part of this study, the panel was comprised of trained evaluators. All methods were carried out in accordance with relevant guidelines and regulations. Informed consent was obtained from all subjects before their participation in the study.

## Data Availability

The data that support the findings of this study are available on request from the corresponding author.
